# Ensemble Modeling of Shifts in the Suitable Distribution and Ecological Niche of the Alpine Tibetan Medicinal Herb *Corydalis hendersonii Hemsl.* Under Climate Change and Human Activity

**DOI:** 10.1002/ece3.73861

**Published:** 2026-06-11

**Authors:** Dehua Wu, Yanlei Liu, Zhixian Jing, Siqi Liu, Ba Qiang, Weiwu Chen, Yiheng Wang, Chuanzhi Kang, Zekun Zhang

**Affiliations:** ^1^ State Key Laboratory for Quality Assurance and Sustainable Use of Dao‐di Herbs, National Resource Center for Chinese Materia Medica China Academy of Chinese Medical Sciences Beijing China; ^2^ School of Landscape and Ecological Engineering Hebei University of Engineering Handan China; ^3^ Tibet Qizheng Tibetan Medicine Co. Ltd. Linzhi China; ^4^ Institute of Chinese Materia Medica China Academy of Chinese Medical Sciences China

**Keywords:** BIOMOD2, climate change, *Corydalis hendersonii*, human activities, Tibetan medical

## Abstract

The Qinghai–Tibet Plateau hosts the rare alpine Tibetan medicinal herb *Corydalis hendersonii* Hemsl. The wild resources of *C. hendersonii* are being increasingly threatened by climate warming and intensified human activity. We employed a biomod2 ensemble species distribution model, integrating climatic variables, light/radiation variables, soil‐property layers, topographic variables, and the Human Footprint Index with 75 spatially rarefied occurrence records, to predict the potential geographic distribution and shifts in habitat suitability under SSP126 and SSP585 (2050s, 2070s, and 2090s), climate dependence, human‐footprint effects, and ecological niche dynamics. 10 algorithms were calibrated with pseudo‐absences and repeated resampling, and the high‐performing models (ROC > 0.9; TSS > 0.8) were combined into a weighted ensemble; XGBoost yielded the highest single‐model accuracy. Ultraviolet radiation (UV‐B) was the dominant predictor of suitability, followed by elevation and key thermal‐contrast variables (BIO1, BIO4, and BIO7), indicating strong adaptation to high‐altitude extreme environments characterized by intense radiation, low mean temperature, and large temperature amplitudes. The current high‐suitability habitats are concentrated in Tibet, with limited patches in southern Xinjiang, southern Qinghai, western Sichuan, and northern Yunnan, reflecting a narrow alpine niche. Future projections diverge strongly between pathways: under SSP126, moderate–high suitability is maintained and slightly expanded, whereas under SSP585, high‐suitability areas contract, leading to an overall shift toward higher‐elevation refugia and a northwestward migration of the centroid. When the Human Footprint Index layer was included, high‐suitability areas consistently decreased and peripheral contraction became more pronounced, indicating that climate‐only projections overestimate realized habitat area. Climatic niche overlap remained high overall but declined and fluctuated more under SSP585, suggesting niche displacement under intense climate warming. The results provide a basis for conserving *C. hendersonii* by prioritizing core habitats and implementing targeted management under future climate change scenarios.

## Graphical abstract

*Corydalis hendersonii*, a rare alpine Tibetan medicinal herb, faces increasing threats from climate warming and intensified human activity. Using a biomod2 ensemble species distribution model that integrates climate variables and the Human Footprint Index, we predict that suitable habitats will remain stable or slightly expand under SSP126 but contract markedly under SSP585, with shifts toward higher‐elevation refugia and northwestward migration. These results highlight the importance of prioritizing core alpine habitats and incorporating human disturbance into conservation planning under future climate change.

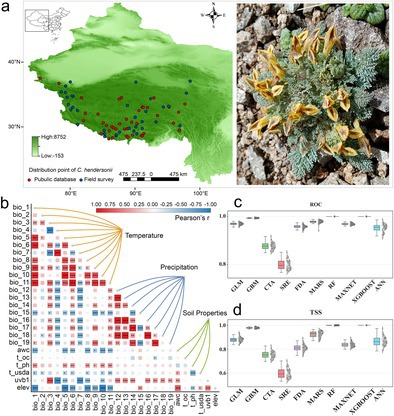

## 1 Introduction

*Corydalis hendersonii* Hemsl. (Papaveraceae), a perennial herb in the genus *Corydalis*, is distributed in high‐elevation mountains and alpine scree habitats in Tibet, Qinghai, and Xinjiang, China. The species represents a characteristic medicinal plant of the plateau region (Li et al. 2020). Classical Tibetan medical texts describe this herb as having effects that include clearing of heat and detoxifying, cooling the blood, alleviating diarrhea, and reducing blood pressure (Bai et al. 2017). Modern pharmacological and phytochemical studies have demonstrated that *C. hendersonii* is rich in alkaloids and other bioactive compounds, exhibiting anti‐inflammatory, analgesic, and cardiovascular protective activities, as well as effects against inflammation‐related diseases and cardio−/cerebrovascular disorders (Shang et al. 2014; Wang et al. 2024). With the ongoing warming and the development of Tibetan medicine, many widely used and culturally important medicinal resources have been subject to unregulated harvesting (Niu et al. 2021; Yang et al. 2024). As an endemic alpine medicinal resource with both ecological and therapeutic value, the status of wild populations and their dynamics under environmental change are directly related to the sustainable utilization of Tibetan medicines and to public health in plateau areas (Ge et al. 2023; Li et al. 2020).

The QTP is one of China's richest regions for medicinal plants, harboring more than 2000 species (Yang et al. 2024). With the ongoing warming and the development of Tibetan medicine, many widely used and culturally important medicinal resources have been subject to unregulated harvesting (Niu et al. 2021; Yang et al. 2024). The Qinghai–Tibet Plateau (QTP) is among the regions most sensitive to global climate change. Warming of the QTP has exceeded the global average, driving rapid changes in the structure and function of alpine ecosystems (Liu and Chen 2000; Yang, Jia, and Hua 2025). In recent decades, climate warming, shifts in precipitation regimes, and intensified human activities, including infrastructure construction, grazing, and tourism, have reshaped the plant diversity of the plateau, threatening endemic alpine species with narrow ranges and specialized niches (Li et al. 2024). Many alpine species are being affected by warming, leading to elevational range shift and changes in community composition. For example, in the Himalayan region, about 87% of alpine plant species have demonstrated upward shifts of their distribution limits, increasing by an average of ~27 m per decade (Telwala et al. 2013). Existing studies suggest that medicinal plants on the QTP and beyond are undergoing habitat loss and population decline under the dual pressures of climate change and human disturbance, thereby threatening their sustainable use (Li and Zhou 2025; Mykhailenko et al. 2025; Zhao et al. 2025). Against this backdrop, systematically evaluating the suitability patterns of alpine medicinal plants under combined climatic and anthropogenic drivers and quantifying the intensity of human impacts are of evident practical importance for developing conservation strategies.

Species distribution models (SDMs) provide powerful tools for elucidating species–environment relationships, predicting potential suitable areas and their responses under future climate scenarios. SDMs have been widely applied in medicinal‐plant assessments and conservation‐priority mapping (Yang et al. 2024; Li and Zhou 2025). Earlier SDM studies often relied on single algorithms, most commonly MaxEnt, with other approaches such as GLM, MARS, ANN, and RF also frequently adopted (Lopatin et al. 2016; Wang, Sun, et al. 2022; Wang et al. 2023). However, single‐algorithm models are sensitive to sampling bias, algorithmic assumptions, and parameter choices, and may overfit the data, leading to substantial uncertainty across datasets and scenarios (Hao et al. 2019; Zhang et al. 2024). Consequently, ensemble forecasting that integrates outputs from multiple algorithms has gained increasing attention. By combining the complementary strengths of various methods, ensembles can improve predictive reliability, reduce single‐model uncertainty, and enhance overall accuracy (Wang, Shi, et al. 2022). The ensemble approach is particularly appropriate for niche‐restricted alpine medicinal plants because it yields confidence intervals and uncertainty estimates that are essential for robust risk assessment. In this context, ensemble frameworks such as the R package Biomod2 have become widely used. Biomod2 integrates multiple algorithms (GLM, GBM, RF, and MaxEnt) and synthesizes their predictions through weighted averaging, thereby increasing accuracy and robustness while reducing the influence of extreme predictions from any single model (Ghehsareh Ardestani and Heidari Ghahfarrokhi 2021; Su et al. 2025). The selected algorithms represent complementary statistical and machine‐learning approaches (Yang, Chen, et al. 2025), allowing the ensemble framework to better characterize nonlinear species–environment relationships and reduce uncertainty associated with individual models. Comparative studies have indicated that, especially when sample sizes are small or environmental gradients are complex, ensemble models generally outperform single MaxEnt models in both predictive performance and uncertainty control, providing a more reliable pathway for assessing suitable habitats of vulnerable species under climate change (Luo et al. 2017). So, such integration is especially advantageous for alpine medicinal plants, which often exhibit fragmented distributions, limited occurrence records, and high environmental heterogeneity. SSP126 and SSP585 were selected because they represent contrasting low‐ and high‐emission scenarios, respectively, thereby covering a broad range of potential future climate conditions on the QTP. In addition, the “ecospat” package can further support analyses of niche dynamics across scenarios, including the effects of human activities (ecospat, n.d.). Together, these tools provide a comprehensive and robust analytical framework for studying ecological processes and predicting species distributions.

Previous research on *C. hendersonii* has focused on phylogeography and functional ecology to describe its distribution patterns and environmental adaptation on the QTP (Li et al. 2020, 2021). SDM‐based assessments remain limited, and the incorporation of human pressures, the high‐elevation UV radiation gradient, and soil physicochemical properties has been relatively minor. Therefore, the present study integrates key predictor variables of climate, soil, UV radiation, and human footprint to predict the distribution of suitable habitat for *C. hendersonii* . We applied Biomod2 ensemble modeling to (1) systematically evaluate the current potential suitable distribution of *C. hendersonii* and identify dominant environmental drivers; (2) quantify spatiotemporal dynamics of suitable habitats and centroid migration trends under SSP126 and SSP585 scenarios and trends in human activity, and (3) combine niche overlap and niche‐shift analyses to explore climatic‐anthropogenic effects on niche stability and potential reconstruction. The findings provide a basis for conservation planning and sustainable utilization of this important alpine Tibetan medicinal resource under ongoing global warming.

## 2 Materials and Methods

### 2.1 Species Geographical Distribution Data

**FIGURE 1:**
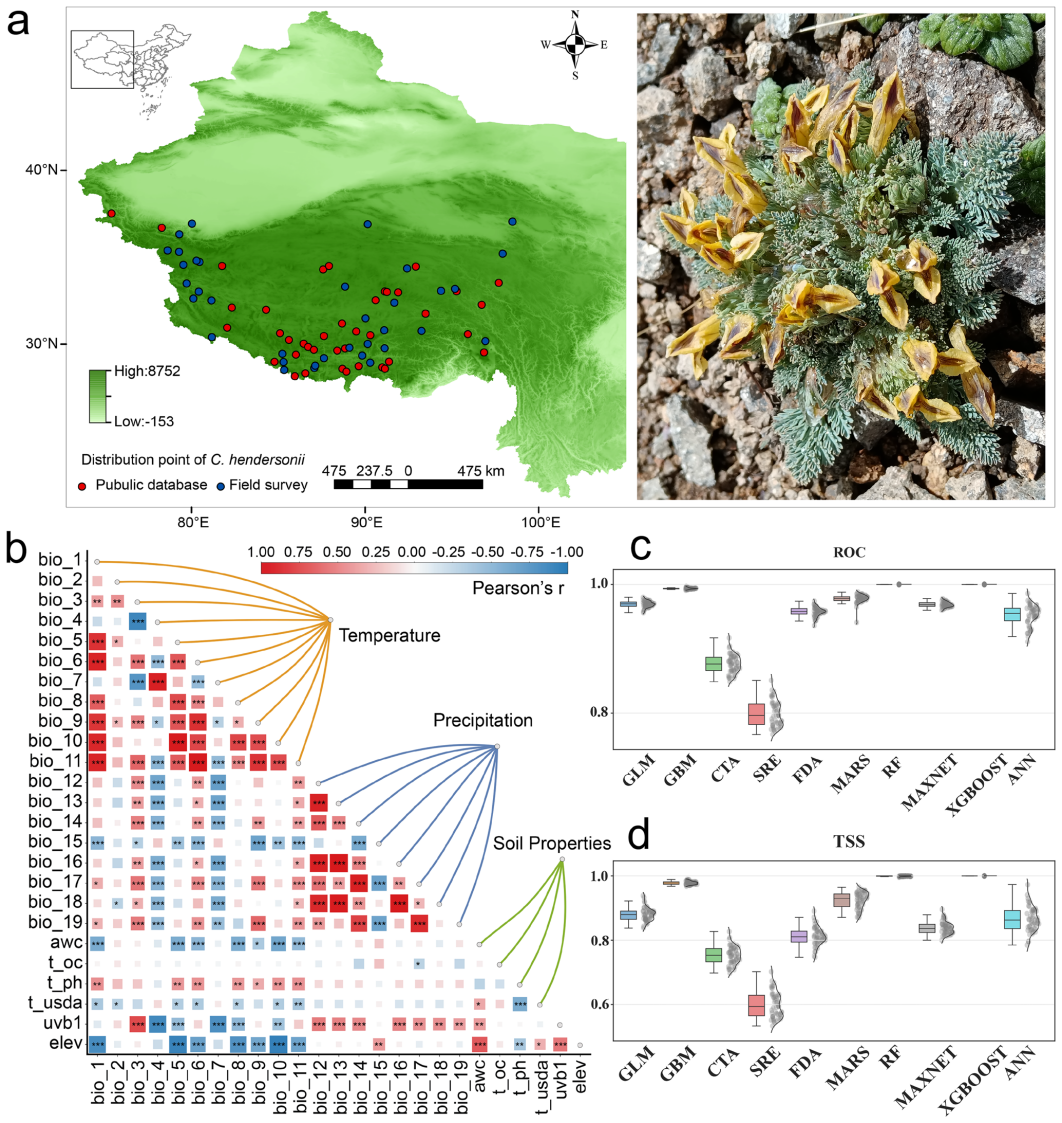
(a) Geographic distribution, plant morphology, and habitat of *C. hendersonii* . (b) Pearson correlations among environmental variables. (c) ROC values for the validation of different models. (d) TSS values for the validation of different models.

Plant occurrence records were compiled from the Global Biodiversity Information Facility (GBIF; https://www.gbif.org/zh/) and the Chinese Virtual Herbarium (CVH; http://www.cvh.ac.cn/). Specimen identifications were first cross‐checked against authoritative floras (e.g., the Flora of China), and records with clear misidentifications were removed. To reduce sampling bias, spatial rarefaction was performed at a 10‐km resolution using SDMtoolbox v2.5. A total of 123 occurrence records were initially collected, of which 75 valid records remained after rarefaction. Among these, 36 records were obtained from GBIF and CVH, while the remaining records were derived from field surveys conducted by our research team (Warren et al. 2010). A total of 123 occurrence records were collected, and after these procedures, 75 valid occurrence records remained; among them, 36 were obtained from GBIF and CVH, whereas the remaining records were derived from field surveys conducted by our research team. These data were saved in CSV format for model construction. The final distribution is shown in Figure 1a.

### 2.2 Analysis of Dominant Environmental Variables

A total of 24 environmental variables were selected for model construction, comprising 19 climatic variables, four soil physicochemical variables, one human influence index, one elevation variable, and one UV variable (Table 1).

**TABLE 1:** The 26 environmental variables of this study.

Variable type	Abbreviation	Environmental variable	Unit
Climatic variables	**BIO1**	Annual mean temperature	°C
	BIO2	Mean diurnal range	°C
	**BIO3**	Isothermality	—
	**BIO4**	Temperature seasonality	CV
	BIO5	Max temperature of the warmest month	°C
	BIO6	Min temperature of the coldest month	°C
	**BIO7**	Temperature annual range	°C
	BIO8	Mean temperature of the wettest quarter	°C
	BIO9	Mean temperature of the driest quarter	°C
	BIO10	Mean temperature of the warmest quarter	°C
	BIO11	Mean temperature of the coldest quarter	°C
	**BIO12**	Annual precipitation	mm
	BIO13	Precipitation of the wettest month	mm
	BIO14	Precipitation of the driest month	mm
	**BIO15**	Precipitation seasonality	CV
	BIO16	Precipitation of the wettest quarter	mm
	BIO17	Precipitation of the driest quarter	mm
	BIO18	Precipitation of the warmest quarter	mm
	BIO19	Precipitation of the coldest quarter	mm
Soil physicochemical variables	awc	Available water capacity per unit soil	mm/m
	texture_usda	USDA soil texture class	—
	org_carbon	Soil organic carbon content	g/kg
	ph_water	Soil pH (in water)	—
Human influence index	hf	Human footprint index	—
Elevation variable	elev	Altitude of occurrence points	m
UV variable	UVB1	UV‐B radiation	J/m^2^/day

*Note:* Variables shown in bold were used in model construction.

All climatic variables were obtained from WorldClim version 2.1 (https://www.worldclim.org/). Current climate layers represent the baseline period of 1970–2000 at a spatial resolution of 2.5′. The *terra* package in R was used to resample all predictors to obtain a consistent spatial resolution (Hijmans et al. 2026). Future climate projections were extracted from the BCC‐CSM2‐MR model for three time slices: the 2050s (2041–2060), the 2070s (2061–2080), and the 2090s (2081–2100). Two Shared Socioeconomic Pathway climate scenarios were considered, SSP126 and SSP585. SSP126 corresponds to a stringent greenhouse gas mitigation pathway, whereas SSP585 represents a high‐emission future with no effective climate policy intervention. These two contrasting scenarios were used to project the future suitable habitats of *C. hendersonii* .

Soil physicochemical data were obtained from the Harmonized World Soil Database (HWSD; https://www.fao.org/soils‐portal/soil‐survey/soil‐maps‐and‐databases/harmonized‐world‐soil‐database‐v12/en/), with an approximate spatial resolution of 1 km (30 arc‐seconds × 30 arc‐seconds), a resolution that is considered suitable for global and regional modeling.

The Human Footprint Index (HFI) layer was incorporated as a key predictor variable for the potential influence of human activities on the distribution of *C. hendersonii* . These data were retrieved from the Socioeconomic Data and Applications Center (SEDAC) of the Earth Observing System Data and Information System (https://sedac.ciesin.columbia.edu/) (Wilson and Jetz 2016). Under current climatic conditions, species distributions were simulated both with and without the human influence variable to evaluate its contribution to present‐day range patterns. Elevation data were sourced from EarthEnv (https://www.earthenv.org/) at a 1‐km resolution. Ultraviolet (UV) radiation data were derived from the glUV database at a spatial resolution of 15 arcminutes (Beckmann et al. 2014).

Environmental values for all 75 occurrence points were extracted using the Extract Multi‐values to Points tool in ArcGIS. Because collinearity among predictors can lead to overfitting in SDMs, variable selection was performed prior to modeling. Pearson correlation coefficients among environmental variables were calculated using the “ggpairs” package in R. Variables with high contributions to the variance were identified, and high‐suitability correlated predictors (|*r*| > 0.7) were removed. When two variables were strongly correlated, the one with the higher contribution was retained. The correlation structure is shown in Figure 1b. The retained climatic variables were then combined with soil physicochemical variables and the human influence index to construct the model, resulting in a final set of 13 predictors.

### 2.3 Construction of SDMs

The current suitable distribution and potential future dynamics of *C. hendersonii* were modeled using the R package “biomod2” (Zou et al. 2024). Ten algorithms implemented in “biomod2” were applied: generalized linear model (GLM), generalized boosting model (GBM), classification tree analysis (CTA), surface range envelope (SRE), flexible discriminant analysis (FDA), multivariate adaptive regression splines (MARS), random forest (RF), maximum entropy model (Maxent), MaxNet (maximum entropy model with lasso regularization, Maxnet), and extreme gradient boosting (XGBoost). For each algorithm, 80% of the occurrence records were randomly selected for training, and the remaining 20% were used for testing. This partitioning strategy helps to reduce overfitting and improve model generalization.

A total of 1000 pseudo‐absence points were randomly generated to better approximate the realized distribution and mitigate biases introduced by uneven spatial sampling. Model calibration was repeated 50 times with resampling to enhance robustness. Pseudo‐absences were generated using a random sampling approach and screened against the environmental background to ensure that they did not overlap with known presence records or violate the ecological assumptions. Model performance was evaluated using the area under the receiver operating characteristic curve (ROC) and the true skill statistic (TSS). ROC values range from 0 to 1, with values > 0.9 indicating excellent performance; TSS values range from −1 to 1, and values > 0.75 are typically considered strong (Wang, Shi, et al. 2022; Nisin et al. 2024). To improve predictive reliability, we adopted a stricter criterion and retained only models with TSS > 0.8 and ROC > 0.9 for ensemble forecasting. The ensemble was generated by weighted averaging, with each model assigned a weight proportional to its evaluation performance; better‐performing models contributed more to the final predictions. The ensemble output thus represents the weighted mean of predictions across all qualifying models.

The ensemble model produced a continuous suitability surface with occurrence probabilities ranging from 0 to 1. For interpretation and mapping, these values were classified in ArcGIS into four suitability classes using equal‐interval breaks: unsuitable (0–0.25), low suitability (0.25–0.50), moderate suitability (0.50–0.75), and high suitability (0.75–1.00).

### 2.4 Measurement of the Ecological Niche of *C. hendersonii*

ENMTools 1.3 was used to quantify pairwise overlap in predicted habitat suitability among model projections under different Shared Socioeconomic Pathway climate scenarios and time periods, using niche overlap metrics including Schoener's *D* and the *I* statistic. Niche overlap was assessed by Schoener's *D* and the Hellinger‐based *I* statistic, metrics that measure the similarity of niches across the three periods and allow comparison of overlap within the native range (Xian et al. 2023). Both *D* and *I* range from 0 (no overlap) to 1 (complete overlap). The effect of climatic differences among periods on the ecological niche was quantified based on the degree of deviation of the *D* and *I* values from 1 (Wang et al. 2026).

To estimate niche breadth and inter‐period overlap, we constructed a two‐dimensional environmental space based on the selected predictors and estimated the species' niche probability distribution within that space. Numeric environmental variables were first standardized to zero mean and unit variance, and principal component analysis (PCA) was used to derive the first two axes (PC1 and PC2) to represent the environmental space (Jolliffe and Cadima 2016). Occurrence coordinates in the PC1–PC2 space were then used to estimate a niche probability surface on a regular grid via Gaussian kernel density estimation (KDE). When the bandwidth was not specified, it was adaptively selected following Scott's rule (Scott 2015). Ecological niche dynamics, including centroid shift, niche overlap, niche unfilling, and niche expansion, were subsequently analyzed in RStudio 4.1.3 using the “ecospat” package based on the PCA‐env framework. Climatic niche similarity tests were conducted with 1000 bidirectional permutations.

### 2.5 Migration of the Centroid of Suitable Areas

Future climate data were obtained from the BCC‐CSM2‐MR model for three future periods: the 2050s (2041–2060), 2070s (2061–2080), and 2090s (2081–2100). Two Shared Socioeconomic Pathway (SSP) scenarios, SSP1‐2.6 and SSP5‐8.5, were used for future projections. The SDM Toolbox v2.5 integrated into ArcGIS was used to calculate the potential geographic centroid of the suitable habitat for *C. hendersonii* across these different time periods and climate scenarios, while jointly accounting for human activity and natural environmental conditions. We visualized the trajectory of centroid shifts by comparing the centroids among scenarios. Changes in distributional area were quantified using the “Distribution changes between binary SDMs” tool in SDM Toolbox v2.5, with a suitability threshold set to > 0.75.

For the range‐change analysis, a threshold of 0.75 was adopted to specifically track changes in high‐suitability habitats. Areas predicted to be high‐suitability under current conditions but not high‐suitability in the future were classified as loss areas. Conversely, areas not currently high‐suitability but projected to become high‐suitability were defined as gain areas. Regions remaining high‐suitability across time periods were categorized as unchanged. The loss and gain ratios were calculated, and the absolute difference between the ratios was used to represent the overall rate of range change. The spatiotemporal dynamics of the potential distribution of *C. hendersonii* under different climate scenarios were visualized in ArcMap 10.8.

### 2.6 Statistical Analyses

Data processing and visualization were primarily conducted in Python. The relationships between *C. hendersonii* and climatic variables were characterized using spline fitting. First, the suitability maps were partitioned in ArcGIS 10.8 (Spatial Analyst Tools) at the resolution of the suitability surface, after which suitability values and environmental information were extracted for 533,467 grid points. After removing outliers and invalid records, each climatic variable was evenly binned into 100 intervals. The mean suitability and sample size were calculated for each bin; those with fewer than 1000 samples were treated as missing to reduce sparsity‐related bias. A univariate spline using the bin centers as the independent variable and mean suitability as the dependent variable was fitted to generate smoothed response curves, and 1000 interpolated points were produced across the full range. Finally, overlaid plots of the raw scatter points and fitted curves were created for subsequent analyses. All computations and plotting were implemented using the NumPy, pandas, SciPy, statsmodels, scikit‐learn, and Matplotlib Python packages.

## 3 Results

### 3.1 Current Geographical Distribution and Model Performance

Among the 10 algorithms evaluated in this study, GLM, GBM, MARS, MAXNET, ANN, XGBoost, and RF all achieved ROC and TSS values greater than 0.8, with XGBoost showing the highest accuracy (Figure 1c,d). The ensemble model (EM) for *C. hendersonii* yielded ROC and TSS values of 0.993 and 0.938, respectively. Although these ensemble metrics were slightly lower than those of XGBoost and RF, this does not indicate inferior performance. The EM emphasizes robustness and generalizability, potentially trading a small amount of peak accuracy for more reliable predictions. By integrating multiple algorithms, the ensemble reduces the risk of overfitting inherent in the use of single models and mitigates biases or extreme predictions from any one method.

**FIGURE 2:**
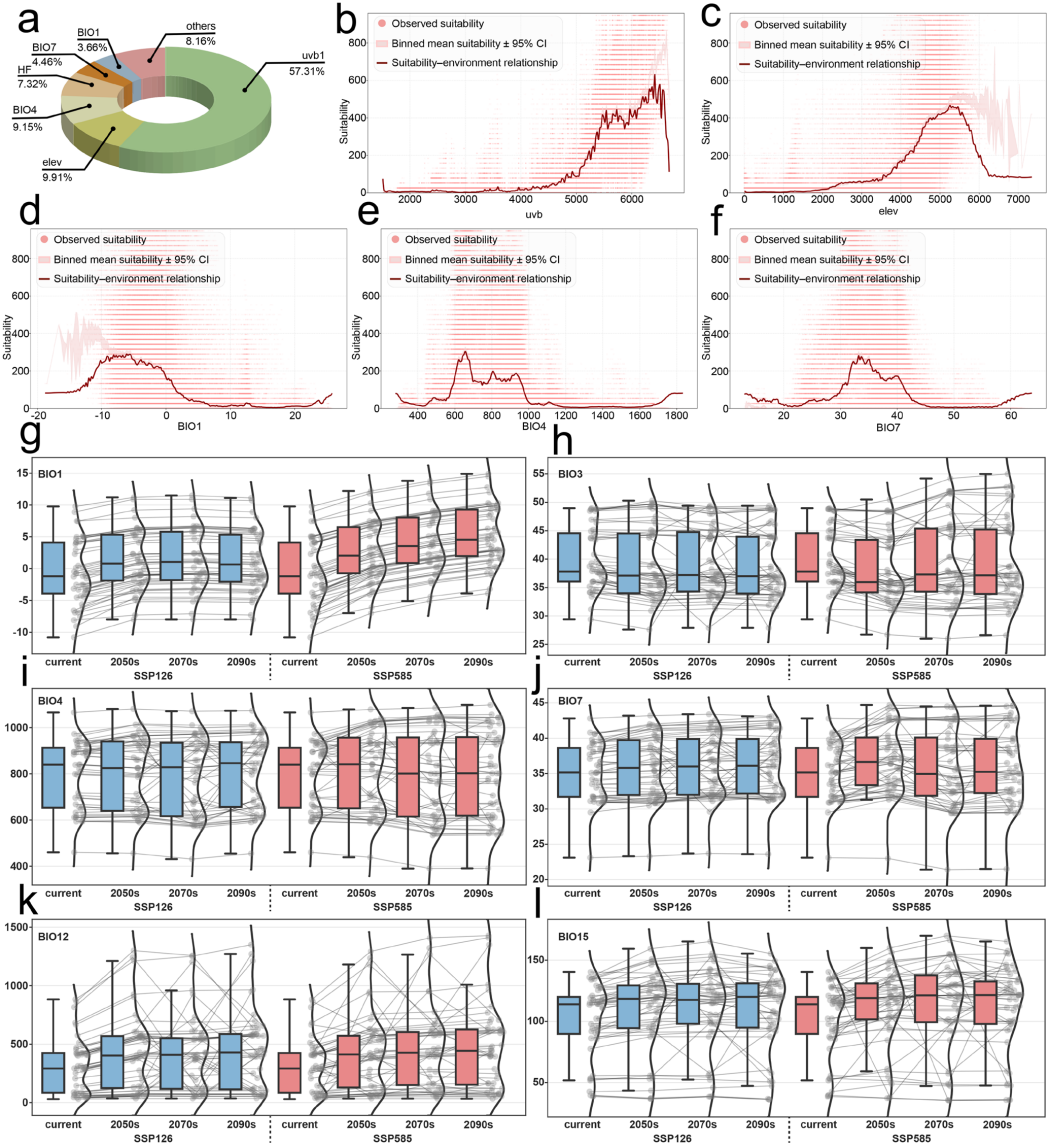
(a) Relative importance (contributions) of environmental predictors in the ensemble model. (b–f) Spline‐fitted response curves between ecological variables and habitat suitability. (g–l) Temporal trends of the bioclimatic variables used in modeling at occurrence sites for *Corydalis hendersonii* under different climate scenarios.

The Biomod2 output identified ultraviolet radiation (UV) as the most important predictor among the selected environmental variables (Figure 2a), followed by elevation (elev). Because UV intensity generally increases with altitude, these two factors are closely linked. The response curves indicate that *C. hendersonii* is adapted to environments with intense UV radiation; within the 95% confidence interval, maximum suitability occurred at approximately 5800 J/m^2^/day (Figure 2b). The high‐suitability areas were concentrated at elevations around 4500 m, higher than those preferred by most plant species (Figure 2c).

Among the six bioclimatic variables retained for modeling, Annual Mean Temperature (BIO1), Temperature Seasonality (BIO4), and Temperature Annual Range (BIO7) contributed the most to model performance. Peak suitability for *C. hendersonii* was observed at an Annual Mean Temperature of about −5°C (Figure 2d), a Temperature Seasonality value near 650 (Figure 2e), and a Temperature Annual Range of roughly 33°C (Figure 2f). These patterns suggest that alpine, cold environments with significant thermal contrast are suitable habitats for *C. hendersonii* . The relationships between suitability and the remaining predictor variables are shown in Figure S1.

Consistent with these environmental preferences, herbarium specimens and literature records showed that *C. hendersonii* exhibited a restricted distribution in China, primarily occurring in Tibet, Qinghai, and adjacent regions. The latitudinal range spanned 28.40°–36.94°N and its longitudinal range 78.28°–98.49°E, indicating that the species predominantly inhabits cold mid‐latitude environments. The reported elevational range was 2219–5979 m, and occurrence records showed that the species is almost exclusively found above 3000 m (Figure 1a).

We further examined the changes in the modeled bioclimatic variables at occurrence sites under different climate scenarios. BIO1 and BIO15 were projected to increase slightly under SSP126 but rise substantially under SSP585 (Figure 2g,l). BIO3 would decline below current levels by the 2090s under SSP126, whereas it would increase continuously under SSP585 (Figure 2h). In contrast, BIO4, BIO7, and BIO12 showed only minor changes across scenarios (Figure 2i–k). Temperature‐related variables that contribute significantly to the distribution of *C. hendersonii* are therefore expected to be markedly affected by high‐emission pathways, implying that unchecked carbon emissions could substantially alter the future distribution of this species.

### 3.2 Predicted Suitable Areas for *C. hendersonii* Across Periods Under Machine‐Learning Models

**FIGURE 3:**
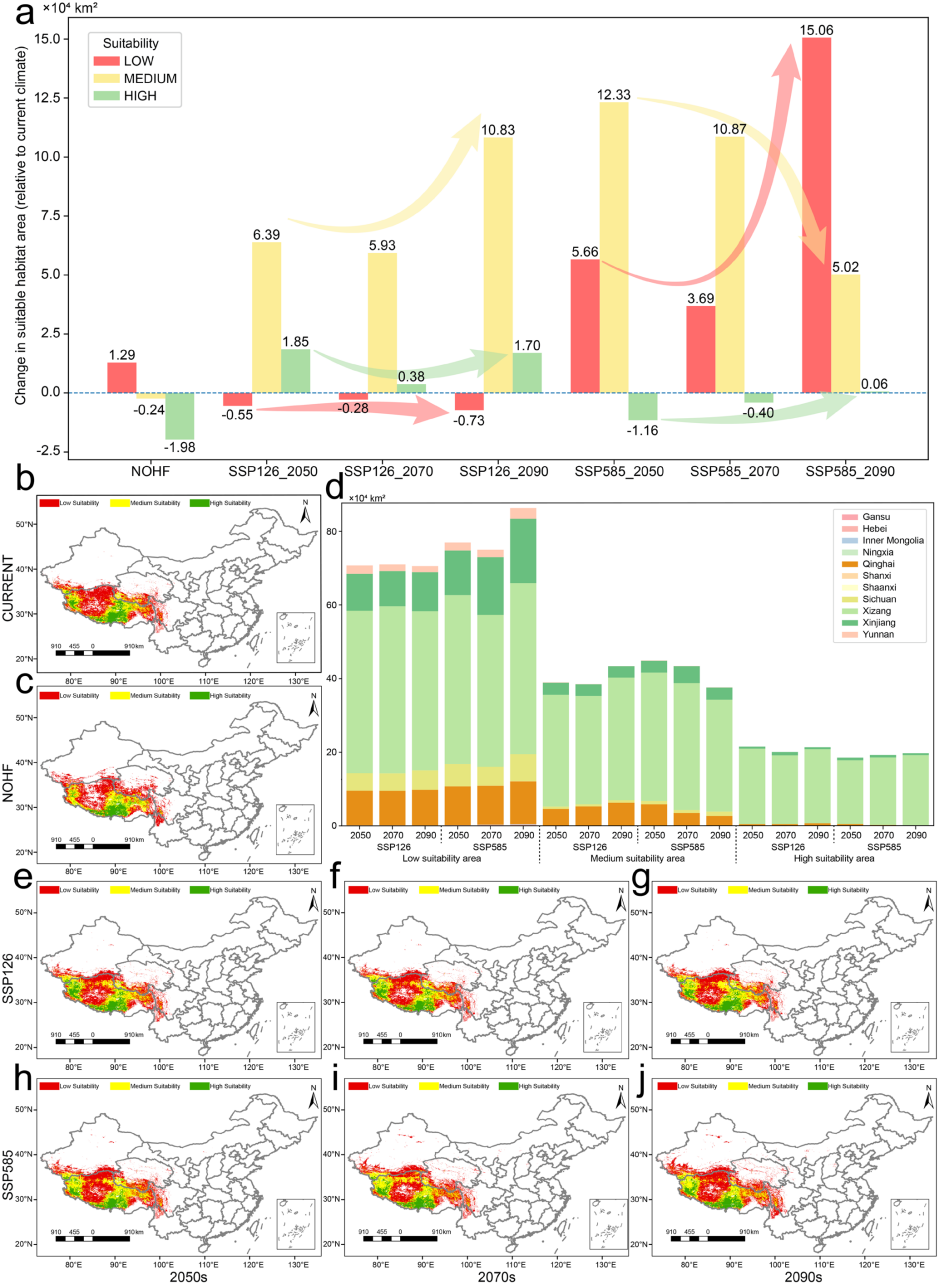
(a) The changes in the areas of high‐, moderate‐, and low‐suitability classes across future time periods and climate scenarios relative to the current baseline. (b) Potential distribution of *Corydalis hendersonii* under the current scenario. (c) Potential distribution of *C. hendersonii* under the scenario excluding human‐activity layers. (d) Areas of high‐, moderate‐, and low‐suitability habitats by province under different climate scenarios and time periods. (e–j) Projected potential distributions of *C. hendersonii* across time periods under various climate scenarios.

Under current conditions, the potential distribution of *C. hendersonii* is concentrated in Tibet, with additional suitable habitats in southern Xinjiang, southern Qinghai, western Sichuan, and northern Yunnan (Figure 3b). This clustered and relatively limited range indicates that the species has stringent environmental requirements and a narrow niche breadth. When the human‐activity layer is excluded, the predicted extent of high‐suitability habitat decreases, and the low‐suitability zone is shifted (compare Figure 3b,c). This pattern suggests that human activities significantly affect the potential distribution of *C. hendersonii* . Therefore, the human footprint layer was retained as a predictor variable in all subsequent projections under different SSP scenarios.

To visualize how different emission trajectories affect future habitat suitability, we quantified changes in suitability‐class areas under each climate scenario relative to the current baseline (Figure 3a). After accounting for human influence, the high‐suitability and moderate‐suitability areas decreased by 1.98 × 10^4^ km^2^ and 0.24 × 10^4^ km^2^, respectively, relative to the current scenario, indicating that human activities may have a negative effect on the potential distribution of *C. hendersonii* .

Under the low‐emission pathway (SSP126), moderate‐suitability and high‐suitability areas are projected to gradually increase. By the 2090s, the moderate‐suitability area would increase by 10.83 × 10^4^ km^2^, and the high‐suitability area by 1.70 × 10^4^ km^2^ relative to the current period, whereas the low‐suitability area would decline. In contrast, projections under the high‐emission pathway (SSP585) are less optimistic. High‐suitability habitat would decrease relative to the current baseline value. Although moderate‐suitability habitat could initially expand compared to current conditions, continued warming would drive a sharp reduction to 5.02 × 10^4^ km^2^, accompanied by a substantial increase in low‐suitability habitat to 15.06 × 10^4^ km^2^ (Table S1).

To clarify regional‐scale changes in suitability, we further examined projected shifts across provinces, municipalities, and autonomous regions (Figure 3d; Table S2). Across all climate scenarios, Tibet consistently retains the largest area of high‐suitability habitat. However, changes in surrounding regions more clearly reflect range dynamics. Qinghai shows the greatest contrast: under SSP126, high‐suitability habitat in Qinghai would reach 0.61 × 10^4^ km^2^ by the 2090s, whereas under SSP585 the area declines to only 0.05 × 10^4^ km^2^, equivalent to approximately 0.005% of China's land area. A similar pattern occurs in Xinjiang, where high‐suitability habitat in the 2090s is projected to be 0.58 × 10^4^ km^2^ under SSP126 but contracts to 0.50 × 10^4^ km^2^ under SSP585, corresponding to approximately 0.060% and 0.052% of China's land area, respectively. Although high‐suitability areas in Yunnan and Sichuan show only slight changes, both moderate‐suitability and low‐suitability areas show substantial declines. In Tibet, the core region, the total area of high‐suitability habitat is projected to be 20.07 × 10^4^ km^2^ in the 2090s under SSP126, but only 19.07 × 10^4^ km^2^ under SSP585, indicating a significant contraction under the high emissions scenario. The spatial patterns of potentially suitable habitats across periods and scenarios are presented in Figure 3e–j.

### 3.3 Contraction and Expansion of Suitable Habitat and Centroid Shifts in *C. hendersonii*

**FIGURE 4:**
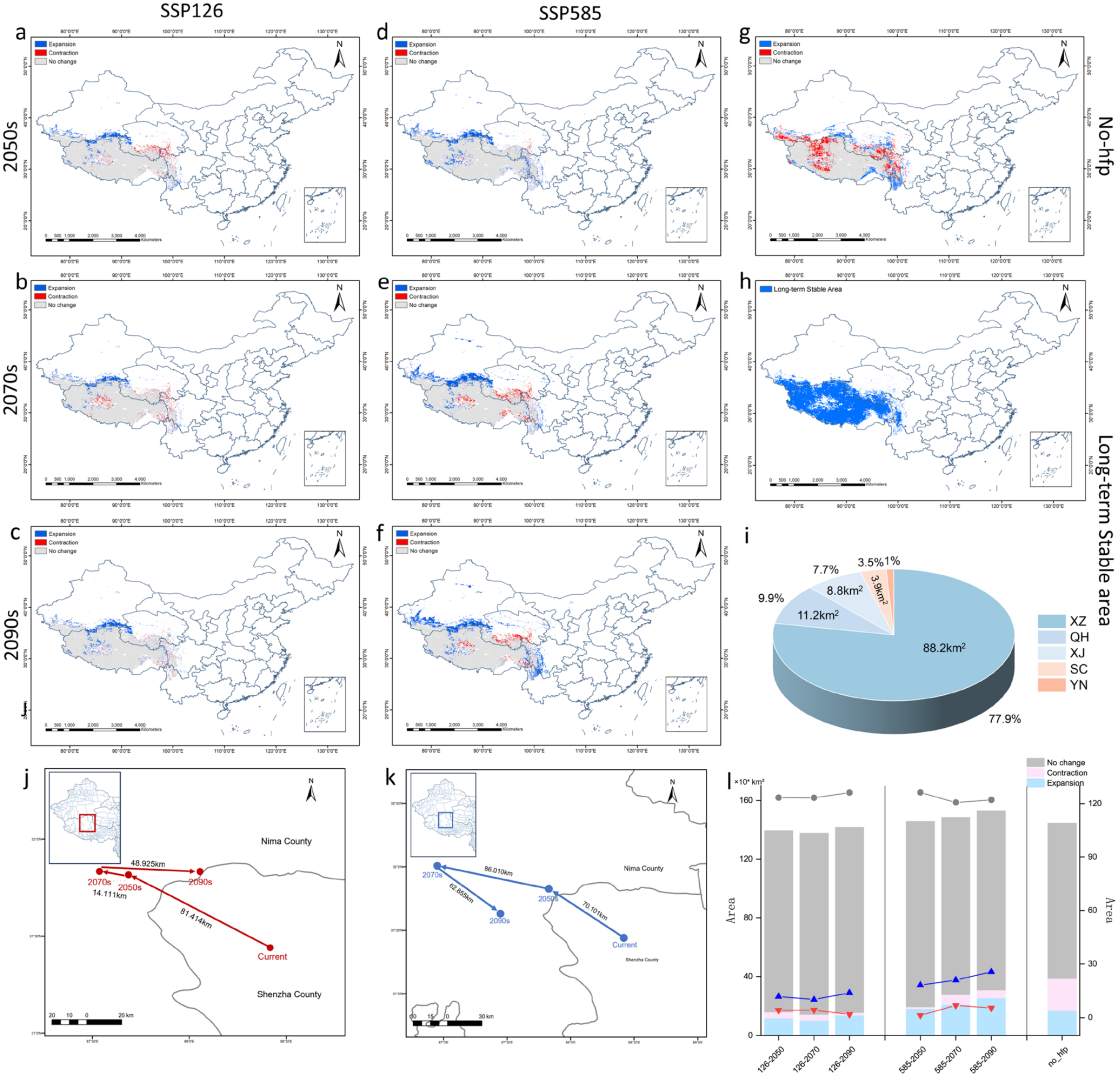
(a–c) Contraction and expansion of suitable habitat for *Corydalis hendersonii* under SSP126. (d–f) Contraction and expansion of suitable habitat for *C. hendersonii* under SSP585. (g) Contraction and expansion of suitable habitat for *C. hendersonii* under the scenario excluding the human‐activity layers. (h) Long‐term stable (unchanged) areas for *C. hendersonii* across all climate scenarios. (i) Proportional contributions of long‐term stable areas by province. (j) Centroid shifts of suitable habitat for *C. hendersonii* under SSP126. (k) Centroid shifts of suitable habitat for *C. hendersonii* under SSP585. (l) Temporal trends in contraction and expansion areas of *C. hendersonii* under different climate scenarios.

After projecting the potential distribution of *C. hendersonii* under different climate scenarios, we quantified spatial transitions in suitable habitat across future periods (Figure 4a–f). Under SSP126, expansion areas are larger, and contraction areas are smaller, while the stable area remains nearly unchanged (Figure 4l; Table S3). This indicates a steady increase in suitable habitat when carbon emissions are effectively controlled. The projected range under SSP126 shows minor contraction, with gradual expansion toward the eastern flank of the Kunlun Mountains (Figure 4a–c).

In contrast, under SSP585, the stable area decreases markedly, suggesting substantial redistribution of suitable habitat. Meanwhile, areas of both contraction and expansion increase through time, implying an overall range shift rather than a simple gain or loss. A considerable area of suitable habitat on the QTP would contract, while new suitable areas emerge eastward toward the Songpan Plateau and the Hengduan Mountains, and westward across the Kunlun range toward the Pamir Plateau (Figure 4f).

Beyond climatic drivers, human activities have a significant negative influence on suitable habitat. Under the scenarios incorporating human footprint effects, suitable areas in Qinghai, Sichuan, and western Tibet exhibit substantial contraction (Figure 4g). Figure 4h shows the regions predicted to remain suitable (stable areas) for *C. hendersonii* across all climate scenarios. Long‐term stable habitats are primarily located in Tibet, Qinghai, Xinjiang, Sichuan, and Yunnan, with Tibet accounting for the largest share, 77.9% of the total stable area (Figure 4i).

We next used ArcGIS spatial analysis to examine shifts in the centroid (the geometric center) of suitable habitat under climate change. Under the low‐emission SSP126 pathway, the centroid first moves northwest by 81.414 km to eastern Nima County, then continues a short westward shift, and subsequently migrates eastward toward the boundary of Shenzha County as emissions remain controlled (Figure 4j). By the 2090s, the centroid stabilizes to the northwest of the current centroid. Under the high‐emission SSP585 pathway, the centroid shifts northwest by 70.101 km to Nima County in the current‐to‐2050s transition, continues northwest by 86.010 km to central Nima City by the 2070s, and finally moves southeast by 62.855 km. Overall, centroid migration is more extensive under high emissions, underscoring SSP585's more substantial impact on future habitat redistribution of *C. hendersonii* .

**FIGURE 5:**
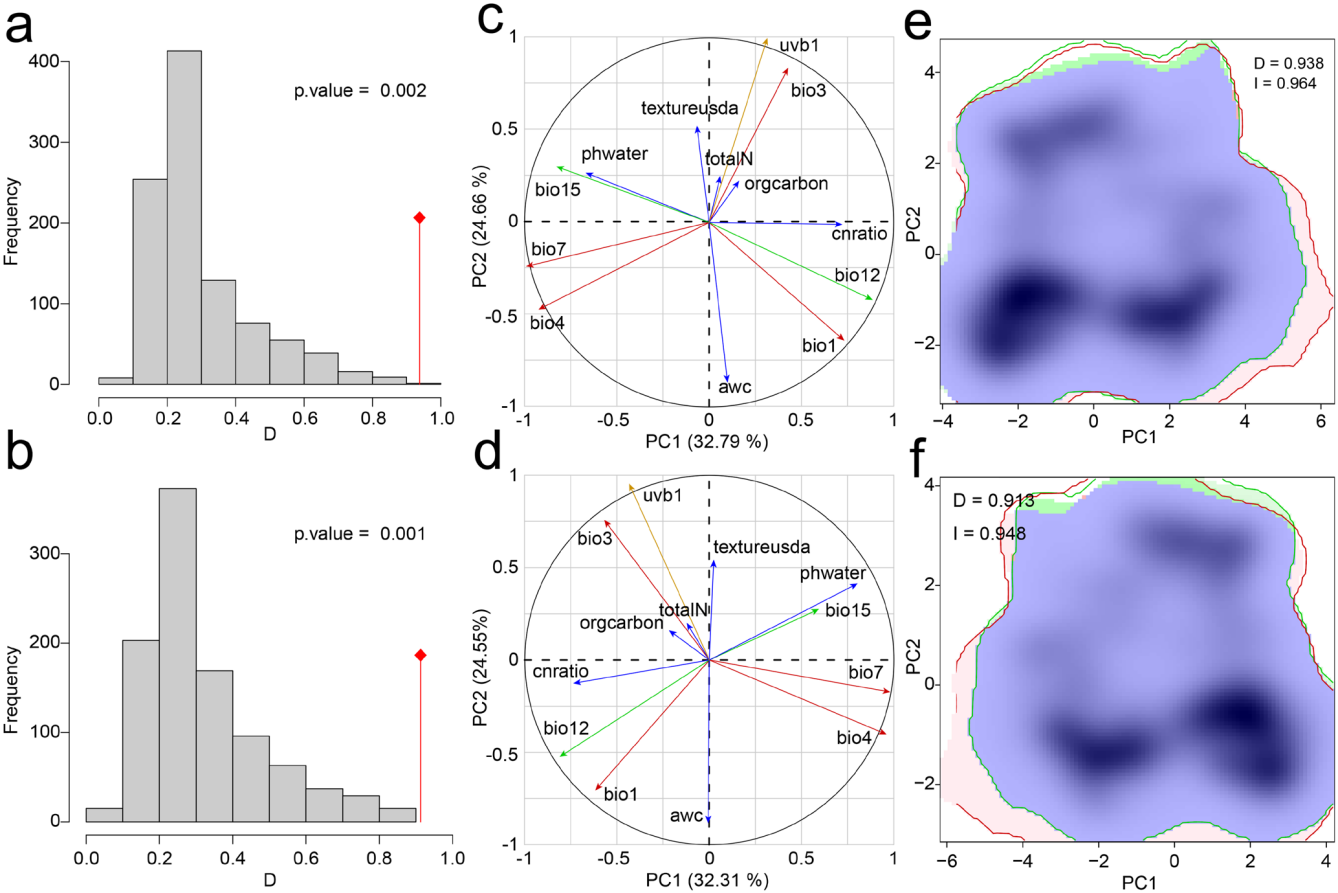
(a) Ecological niche similarity across pathways in the 2090s under SSP126. (b) Ecological niche similarity across pathways in the 2090s under SSP585. (c, d) PCA loading plots for principal climatic variables shaping ecological niches; the arrow colors denote variable classes: Red: Temperature, blue: Soil, green: Precipitation, orange: Radiation. (e, f) Ecological niches of *Corydalis hendersonii* under SSP126 and SSP585.

Figure 5 illustrates the modeled climatic niche dynamics of *C. hendersonii* across different carbon‐emission pathways and time periods. Because all comparisons involve the same species, climatic niches among periods and scenarios are expected to show substantial overlap; accordingly, Schoener's *D* remains generally high. Against this backdrop, we focus on subtle shifts in *D* to quantify how different periods or emission pathways will affect niche characteristics.

In the projections for the 2090s, climatic niches under SSP126 and SSP585 still have significant overlap (Figure 5a,b), and niche similarity tests indicate no overall difference between the two pathways. The PCA‐based environmental‐space projections (Figure 5c,d) provide a complementary perspective by visually depicting the changes in niche position and shape, and by clarifying how multiple climatic predictors jointly structure the niche across pathways and periods. Furthermore, although the overall overlap between periods remains high, niche overlap with the current climate is comparatively lower, and D fluctuates more strongly under the high‐emission pathway (SSP585) (Figure 5e,f; Figure S2). In contrast, *D* varies only slightly under the low‐emission pathway (SSP126), indicating that effective carbon‐mitigation policies help maintain *C. hendersonii* 's current climatic niche. Overall, climatic niche overlap remains high across pathways and periods, but small increases or decreases in *D* illustrate the impacts of temporal change and differing intensities of human‐driven climate change on the species' niche.

## 4 Discussion

### 4.1 Major Environmental Drivers of the Potential Distribution of *C. hendersonii*

By integrating multiple niche‐based modeling approaches, this study has examined the present and future potential distribution of the rare endemic plant *C. hendersonii* and identified the key environmental mechanisms underlying its range dynamics. The environmental variables used in this study represent different ecological dimensions that may constrain the distribution of *C. hendersonii* , including energy and thermal conditions, water availability, edaphic properties, topographic gradients, radiation exposure, and anthropogenic disturbance (Xian et al. 2023; Wang, Guo, et al. 2025). Among these variables, ultraviolet radiation and elevation were the dominant predictors, suggesting that high‐elevation environmental conditions play an important role in shaping the distribution of *C. hendersonii* . Temperature‐related variables, particularly Annual Mean Temperature, Temperature Seasonality, and Temperature Annual Range, further indicate that the species is adapted to cold alpine environments with large seasonal thermal fluctuations (Xie et al. 2019). In contrast, precipitation and soil variables may influence habitat suitability by regulating moisture availability (Zuquim et al. 2020) and substrate conditions, although their relative contributions were lower than those of radiation, elevation, and temperature. The Human Footprint Index provides an additional constraint by capturing anthropogenic pressures such as grazing, infrastructure development, tourism, and medicinal plant harvesting, which may reduce the availability of otherwise climatically suitable habitats (Sofi et al. 2022). Therefore, the predicted distribution of *C. hendersonii* should be interpreted as the combined outcome of climatic filtering, high elevation environmental stress, substrate‐related habitat conditions, and human disturbance.

The model outputs consistently indicated that ultraviolet radiation (UV) was the most influential environmental determinant of *C. hendersonii* 's distribution. Conventional SDMs typically emphasize climatic variables such as temperature and precipitation (Chen et al. 2024), whereas the inclusion of UV in our framework reveals the prominent role of non‐climatic factors in structuring the distribution of high‐elevation taxa. The strong association between UV intensity and elevation suggests that *C. hendersonii* is adapted to the composite environmental conditions characteristic of alpine habitats, including intense radiation and large thermal amplitudes. Habitat suitability peaked at approximately 5800 J/m^2^/day UV, suggesting that the species may possess adaptive physiological mechanisms that facilitate tolerance to strong UV exposure in alpine environments (Shi and Liu 2021). Such adaptations may enable the species to occupy harsh microhabitats, such as alpine screes and sparsely vegetated rocky substrates where environmental conditions are relatively severe.

Among the temperature‐related bioclimatic variables, Annual Mean Temperature (BIO1), Temperature Seasonality (BIO4), and Temperature Annual Range (BIO7) were the most influential predictors. The highest habitat suitability occurred at an Annual Mean Temperature of approximately −5°C and a Temperature Annual Range of approximately 33°C, indicating that *C. hendersonii* is strongly associated with cold alpine environments characterized by pronounced seasonal and annual thermal variation (Dong et al. 2023). BIO1 reflects the overall thermal regime and may constrain the species' distribution by affecting growth, phenology, photosynthetic activity, and overwintering survival. BIO4 and BIO7 further describe temporal temperature variability and annual thermal amplitude, suggesting that *C. hendersonii* is adapted not only to low mean temperatures but also to strong thermal fluctuations typical of high‐elevation habitats. Therefore, changes in these bioclimatic factors, especially warming‐driven increases in mean temperature, may alter habitat suitability and drive future shifts in the potential distribution of this species.

Collectively, these findings suggest that the distribution of *C. hendersonii* is jointly shaped by high radiation stress, substrate related habitat conditions, and anthropogenic disturbance, reflecting the complex environmental filtering processes characteristic of the QTP.

### 4.2 The Effects of Human Activities on the Distribution of *C. hendersonii* and Its Future Suitability Dynamics

After incorporating the human‐activity layer, the predicted area of high‐suitability habitat for *C. hendersonii* decreased markedly. This provides clear evidence that human disturbance has become a key force shaping the species' realized distribution, comparable in importance to climatic constraints. On the QTP, human impacts are primarily associated with overgrazing (Ye et al. 2025), road and infrastructure development (Behmanesh et al. 2024), tourism (Zhang et al. 2022), and the harvesting of medicinal plants (Tang et al. 2024). These activities can directly degrade or fragment habitats and may also indirectly reduce habitat suitability by altering the soil structure, moisture regimes, and competitive interactions.

This finding has important implications for conservation biology. Theoretical distributions inferred solely from climate factors can substantially overestimate the actual space available for species persistence. The current fragmented distribution of *C. hendersonii* is likely a remnant of its original climatically suitable range, eroded by long‐term human activity. Therefore, any conservation measures aimed at population recovery or assisted migration must include a fine‐scale assessment of human pressures in candidate areas. Otherwise, even climatically optimal regions may fail to support successful introductions or long‐term protection if the level of human disturbance remains high. Our results strongly support the emerging direction of integrating socioeconomic driving factors into biodiversity modeling, and we thus caution that conservation planning that ignores anthropogenic pressure is likely to be detached from ecological reality.

Future projections further suggest that under the low‐emission pathway (SSP126), suitable habitats for *C. hendersonii* can be maintained and may even expand slightly, whereas under the high‐emission pathway (SSP585), its suitable range—especially high‐suitability areas—faces significant contraction. This contrast suggests that reduced climate‐change intensity may help maintain suitable habitats for alpine endemic species, as also reported in previous studies on mountain plant responses to future climate scenarios (Wang, Xu, et al. 2025). Geographic analyses further showed that habitat loss was spatially uneven. Tibet, the core distribution region of *C. hendersonii* in our projections, was predicted to retain a relatively large extent of high‐suitability habitat, whereas marginal regions such as Qinghai and Xinjiang were predicted to show sharp declines in suitability under SSP585, particularly in high‐suitability habitats, with some cases approaching disappearance. This pattern may be consistent with the core–periphery hypothesis, which suggests that peripheral populations are often closer to ecological tolerance limits and therefore more vulnerable to environmental change (Radomski 2026). Nevertheless, this hypothesis is used here only as a possible explanation, because physiological tolerance and population dynamics were not directly examined in this study.

Although peripheral populations may occupy smaller areas, they can harbor unique genetic resources adapted to local conditions. Their loss would directly reduce genetic diversity, thereby weakening the evolutionary potential of the species to cope with future climate changes (Ittonen et al. 2022). Conservation strategies should thus be spatially differentiated. In the Tibetan core area, priority should be given to establishing large, contiguous protected landscapes and minimizing habitat fragmentation driven by human activities. In peripheral regions such as Qinghai and Xinjiang, more proactive interventions are warranted, including ex situ conservation programs and assisted migration to nearby potential refugia that are less exposed to climate change, thereby functioning as “climate shelters” for vulnerable edge populations.

### 4.3 Contraction and Expansion of Potential Suitable Areas and Niche Overlap Under Different Climate Scenarios

In the context of ongoing global warming, alpine ecosystems are widely being recognized as among the most climate‐sensitive regions (Lamprecht et al. 2018; Verrall and Pickering 2020). The suitable habitat for *C. hendersonii* follows sharply contrasting trajectories under SSP126 versus SSP585. In particular, under the high‐emission scenario (SSP585), the total suitable area contracts substantially and shifts toward higher‐elevation regions such as the Kunlun Mountains, the Pamir Plateau, and the Hengduan Mountains. This pattern is consistent with the classic hypothesis of upward and poleward range shifts in response to warming (Ramalho et al. 2023). Importantly, the redistribution observed is not merely a geographic displacement but instead reflects a reconfiguration of the species' niche under climatic stress. As temperatures continue to rise, mid‐ and lower‐elevation habitats that are currently suitable may become increasingly unfavorable, forcing populations to retreat into colder high‐mountain environments. While such shifts may represent a short‐term adaptive response, the long‐term prospects are constrained by the limited extent of high‐elevation habitats, topographic fragmentation, and non‐climatic limitations involving soils, moisture availability, and substrate stability, all of which may ultimately threaten species persistence (Chevalier et al. 2023).

Niche analyses provide a complementary lens for interpreting climate responses. Our results suggest relatively high niche overlap under SSP126, whereas niche overlap declines markedly under SSP585, suggesting niche restructuring under intense warming. This raises a key conceptual issue: does niche “stability” necessarily imply “adaptive capacity” (Zhou et al. 2023)? From an evolutionary‐ecological perspective, niche conservatism is often interpreted as evidence that species have a limited capacity to rapidly adapt to novel environments (Qiao et al. 2024). However, in the context of rapid climate change, niche flexibility may also confer survival advantages. The niche shifts predicted for *C. hendersonii* under SSP585 may represent an emergency response to increasing heat or drought stress, potentially involving attempts to broaden its climatic tolerance. Whether such broadening is sustainable or entails a decline in fitness remains uncertain and should be tested through integrative physiological experiments and population‐genetic analyses.

## 5 Conclusion

This study integrated SDMs and climatic niche analyses to assess the current and potential future distribution of the rare alpine endemic plant *C. hendersonii* on the QTP and to identify major drivers of range change. Occurrence records from GBIF and the Chinese Virtual Herbarium were verified, spatially rarefied, and rasterized, yielding 75 reliable presences. Twenty‐six predictors (bioclimatic, soil, UV radiation, elevation, and Human Footprint Index) were assembled and reduced to 13 variables after correlation screening. Ensemble SDMs built with 10 algorithms in biomod2 showed excellent performance (ROC > 0.9, TSS > 0.8). Under the current climate, high‐suitability habitat is concentrated in Tibet, with small additional patches in southern Xinjiang, southern Qinghai, western Sichuan, and northern Yunnan, indicating a narrow high‐elevation niche. UV radiation contributed most to the model predictions, followed by elevation and temperature variables (BIO1, BIO4, BIO7), suggesting adaptation to a high‐altitude stress complex characterized by intense radiation, low mean temperature, and large thermal amplitudes. Future projections revealed contrasting trajectories between emission pathways. Under SSP126, moderate‐suitability and high‐suitability areas would gradually expand and remain relatively stable. Under SSP585, high‐suitability habitat would contract markedly; moderate‐suitability habitat would increase initially but then decline, while low‐suitability zones would expand. Incorporating human footprint consistently reduces the area of high‐suitability habitat, indicating substantial anthropogenic limitation and suggesting that climate‐only models overestimate realized habitat. Range‐change and centroid analyses indicate minor contraction and east‐Kunlun expansion under SSP126, but significant losses of stable habitat, upslope shifts toward higher‐elevation refugia, and longer northwestward centroid migrations under SSP585. Climatic niche overlap remains high overall, whereas niche displacement is more substantial under SSP585, implying niche restructuring under intense warming. Overall, the persistence of *C. hendersonii* will depend on joint efforts to mitigate emissions, protect UV‐linked alpine habitats, and reduce local human disturbance. The results provide a practical framework for conservation planning by identifying climatically stable refugia, areas vulnerable to habitat loss, and potential target regions for habitat restoration, long‐term monitoring, and assisted migration of *C. hendersonii* .

## Author Contributions

**Dehua Wu:** formal analysis (equal), methodology (equal), writing – original draft (equal). **Yanlei Liu:** methodology (equal), visualization (equal), writing – original draft (equal). **Zhixian Jing:** methodology (equal), visualization (equal). **Siqi Liu:** data curation (equal). **Ba Qiang:** investigation (equal). **Weiwu Chen:** software (equal). **Yiheng Wang:** data curation (equal), software (equal), writing – review and editing (equal). **Chuanzhi Kang:** conceptualization (equal), project administration (lead), supervision (equal). **Zekun Zhang:** conceptualization (equal), investigation (equal), supervision (equal), writing – review and editing (equal).

## Funding

This research was funded by Science and Technology projects of Xizang Autonomous, China, grant number: No. XZ202402ZD0002; the National Key Research and Development Program of China, grant number: No. 2023YFC3503801; China Agriculture Research System, grant number: No. CARS‐21.

## Conflicts of Interest

The authors declare no conflicts of interest.

## Supporting information


**Figure S1:** The relationships between suitability and the remaining predictor variables.
**Figure S2:** Ecological niche shifts under SSP126 and SSP585 scenarios in the 2050s and 2070s.

**Table S1:** Area of each suitability category (×104 km^2^).
**Table S2:** Distribution of habitat suitability simulation across provinces.
**Table S3:** Changes in area suitability during different pathways and periods (km^2^).

## Data Availability

Data is contained within the article or Supporting Information.
