# Supplementary material for: Ensemble Modeling of Shifts in the Suitable Distribution and Ecological Niche of the Alpine Tibetan Medicinal Herb Corydalis hendersonii Hemsl. Under Climate Change and Human Activity
Source: Ecol Evol. 2026 Jun 11;16(6):e73861. doi: 10.1002/ece3.73861 (PMC13260866; doi:10.1002/ece3.73861)

Figure S1. The relationships between suitability and the remaining predictor variables


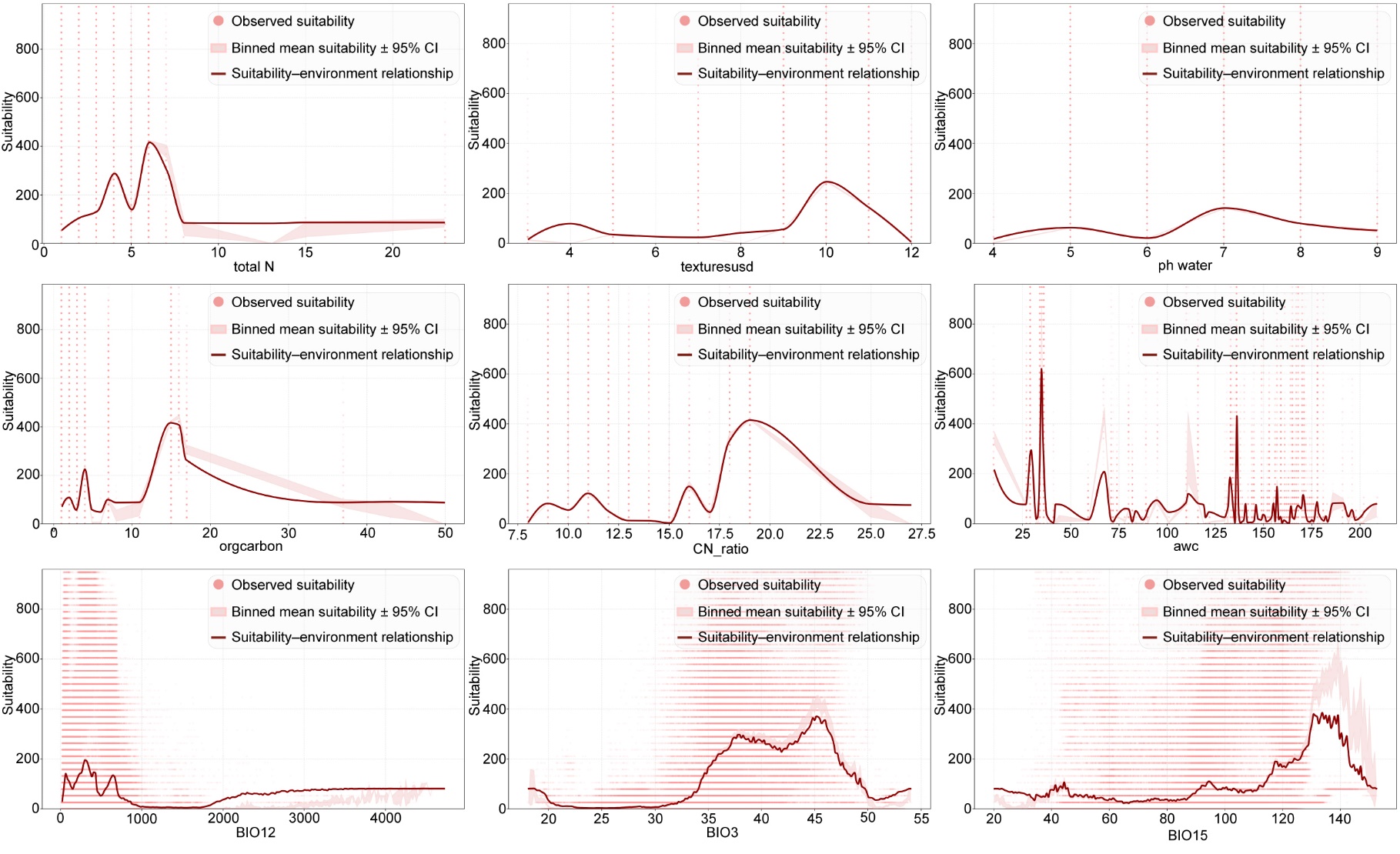


Figure S2. Ecological niche shifts under SSP126 and SSP585 scenarios in the 2050s and 2070s.


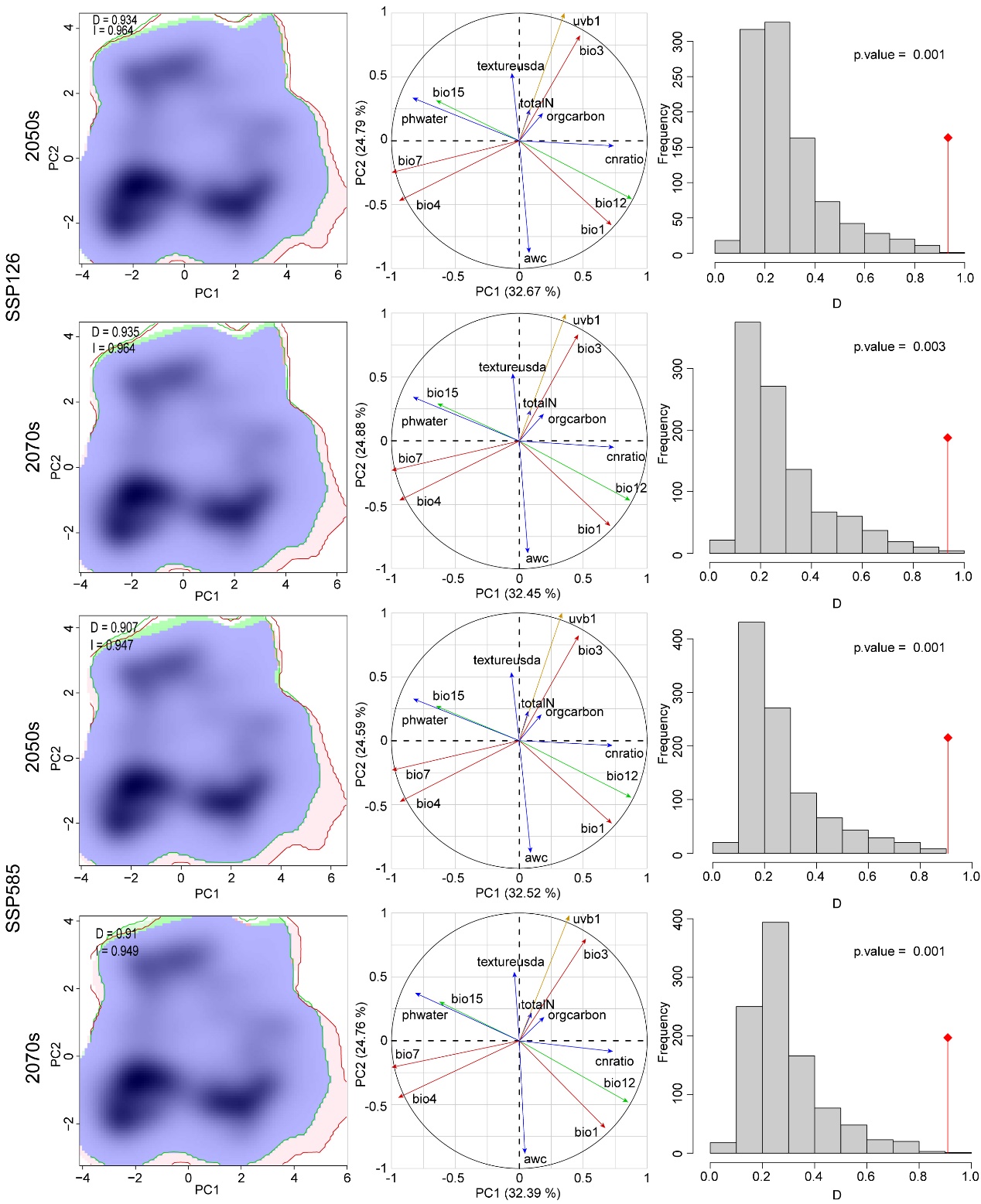

Supplement: Supplementary file 1 — Figure S1: The relationships between suitability and the remaining predictor variables. Figure S2: Ecological niche shifts under SSP126 and SSP585 scenarios in the 2050s and 2070s. [file ECE3-16-e73861-s002.docx]
